# Potential impacts of post-Brexit agricultural policy on fruit and vegetable intake and cardiovascular disease in England: a modelling study

**DOI:** 10.1136/bmjnph-2019-000057

**Published:** 2020-01-14

**Authors:** Paraskevi Seferidi, Anthony A Laverty, Brendan Collins, Piotr Bandosz, Simon Capewell, Martin O’Flaherty, Christopher Millett, Jonathan Pearson-Stuttard

**Affiliations:** 1 Public Health Policy Evaluation Unit, School of Public Health, Imperial College London, London, UK; 2 Department of Public Health and Policy, University of Liverpool, Liverpool, UK

**Keywords:** dietary patterns

## Abstract

**Background:**

Current proposals for post-Brexit agricultural policy do not explicitly incorporate public health goals. The revised agricultural policy may be an opportunity to improve population health by supporting domestic production and consumption of fruits and vegetables (F&V). This study aims to quantify the potential impacts of a post-Brexit agricultural policy that increases land allocated to F&V on cardiovascular disease (CVD) mortality and inequalities in England, between 2021 to 2030.

**Methods:**

We used the previously validated IMPACT Food Policy model and probabilistic sensitivity analysis to translate changes in land allocated to F&V into changes in F&V intake and associated CVD deaths, stratified by age, sex and Index of Multiple Deprivation. The model combined data on F&V agriculture, waste, purchases and intake, CVD mortality projections and appropriate relative risks. We modelled two scenarios, assuming that land allocated to F&V would gradually increase to 10% and 20% of land suitable for F&V production.

**Results:**

We found that increasing land use for F&V production to 10% and 20% of suitable land would increase fruit intake by approximately 3.7% (95% uncertainty interval: 1.6% to 8.6%) and 17.4% (9.1% to 36.9%), and vegetable intake by approximately 7.8% (4.2% to 13.7%) and 37% (24.3% to 55.7%), respectively, in 2030. This would prevent or postpone approximately 3890 (1950 to 7080) and 18 010 (9840 to 28 870) CVD deaths between 2021 and 2030, under the first and second scenario, respectively. Both scenarios would reduce inequalities, with 16% of prevented or postponed deaths occurring among the least deprived compared with 22% among the most deprived.

**Conclusion:**

Post-Brexit agricultural policy presents an important opportunity to improve dietary intake and associated cardiovascular mortality by supporting domestic production of F&V as part of a comprehensive strategy that intervenes across the supply chain.

**What this paper adds**

- Following Brexit, the UK has signalled its intention to shift towards a new agricultural policy. However, current proposals for post-Brexit agricultural policy do not explicitly incorporate public health goals.
- Post-Brexit agricultural policy presents an opportunity to improve domestic production and dietary intake of fruits and vegetables in England, with beneficial impacts on CVD mortality and inequalities.
- To achieve this, support for British-grown fruits and vegetables should be part of a comprehensive agricultural strategy that intervenes across the whole supply chain.

## Introduction

Brexit, the planned exit of the UK from the European Union (EU), will impact UK agricultural policy. While part of the EU, the UK abides by the European Common Agricultural Policy (CAP), which regulates agriculture across EU Member States under a common regime. Post-Brexit, the UK has signalled its intention to shift towards a new agricultural policy, currently outlined in a draft Agriculture Bill introduced in September 2018.^1^ The Agriculture Bill recommends the gradual removal of current CAP payments during a 7-year transition period, between 2021 to 2027. It proposes a new financial assistance system to replace CAP payments, which will be based on the provision of ‘public money for public goods’. These public goods would mainly involve environmental goals (box 1).

Box 1 **Public goods outlined in the Agriculture Bill**

- Managing land or water in a way that protects or improves the environment.
- Supporting public access to and enjoyment of the countryside, farmland or woodland and better understanding of the environment.
- Managing land or water in a way that maintains, restores or enhances cultural heritage or natural heritage.
- Mitigating or adapting to climate change.
- Preventing, reducing or protecting from environmental hazards.
- Protecting or improving the health or welfare of livestock.
- Protecting or improving the health of plants.

Source: Agriculture Bill^1^

The omission of explicit health goals in the Agriculture Bill would appear to be a missed opportunity. Leading public health and farming think tanks in the UK have recommended the consideration of public health as a public good in the new Agriculture Bill,^2 3^ to capture the dual purpose – a sustainable and healthy agricultural policy. The food supply chain begins at the farm; thus, agriculture is a determinant of the food system and shapes diet through supply-side interventions to the food environment.^4^ Therefore, post-Brexit agricultural policy is an opportunity to improve UK diets by shaping the availability, affordability, diversity, quality and marketing of British-grown fruits and vegetables (F&V).^2 3^

F&V intake in the UK is suboptimal – currently only 31% of adults meet the governmental recommendations of five F&V per day.^5^ Low F&V intake is associated with a substantial burden of disease, including cardiovascular disease (CVD).^6 7^ F&V account only for a small proportion of overall agricultural production in the UK, with only 2.7% of total croppable land used for horticultural crops^8^ and domestic production accounting for 16% of fruit and 52% of vegetable total supply in 2018.^8^ Thus, Brexit is planned during a period when there is an urgent need to boost domestic F&V production and consumption in the UK to improve health and reduce inequalities. This research aims to quantify the potential impacts of a post-Brexit agricultural policy that increases domestic production of F&V on CVD mortality and inequalities in England, between 2021 to 2030.

## Methods

We extended the previously validated IMPACT Food Policy model^9 10^ to estimate the potential effect of increasing land allocated to F&V as part of the post-Brexit agricultural regime on production and intake of F&V. Changes in F&V intake were then translated into changes in mortality of coronary heart disease (CHD), ischaemic stroke and haemorrhagic stroke by age group (25 to 34 until 85+ years), sex and quintile of Index of Multiple Deprivation (IMD) 2010, between 2021 to 2030 in England. Stratification by level of deprivation allowed us to quantify the potential impact of modelled scenarios on CVD inequalities. A schematic representation of the model is presented in online supplementary appendix figure A1.

10.1136/bmjnph-2019-000057.supp1 Supplementary data

### Data sources

Model inputs and their sources are presented in table 1.

**Table 1:** Model inputs and data sources

Model input	Data source
Total and F&V agricultural land, 2010–2018 (England)	DEFRA, June Survey of Agriculture^11^
Provisional Agricultural Land Classification (England)	Natural England^14^
F&V yield, estimated using area and production data, 2010–2018 (UK)	DEFRA, Agriculture in the UK^13^
F&V supply, estimated using production, import and export data, 2010–2018 (UK)	DEFRA, Horticulture Statistics^12^
F&V purchases, 2008-2016/2017 (UK)	Family Food module of the Living Costs and Food Survey^16^
F&V waste at household level, 2012 (UK)	Waste & Resources Action Programme^17^
Population projections, England, 2021–2030 (2016-based and mid-year)	O^27^NS
F&V intake by age, sex and IMD, England	National Diet and Nutrition Survey Rolling Programme Years 1–4 and 7–8^28^
RR for CHD/ ischaemic stroke/ haemorrhagic stroke by serving of fruit/vegetable intake by age	Micha *et al*, 2017^19^
CHD and stroke mortality projections for England by age, sex and IMD, 2021–2030	Own estimations using data from the ONS
Impact of No Deal Brexit on F&V intake in England in 2021	Seferidi *et al*, 2019^23^

CHD, coronary heart disease; DEFRA, Department of Environment and Rural Affairs; F&V, fruit and vegetable; IMD, Index of Multiple Deprivation; ONS, Office for National Statistics; RR, relative risk.

We integrated a number of data inputs from the Department of Environment and Rural Affairs. Data on land used for F&V production (% total agricultural land) were obtained from the June Survey of Agriculture,^11^ which collects information from farmers on their agricultural activities on 1^st^ June every year. Data from Horticulture Statistics^12^ provided information on F&V supply (tonnes), estimated as the sum of F&V production and supply excluding exports, whereas data from the annual Agriculture in the UK report^13^ provided an estimate of F&V yield (tonnes/hectare), estimated using data on agricultural area and production.

Modelled scenarios were informed by data on Agricultural Land Classification (ALC) in England.^14^ ALC categorises agricultural land in England into grades of land quality based on three criteria: climate, site and soil.^15^ Grades 1 and 2 are of the highest quality, allowing to grow a wide range of horticultural crops, including F&V. Grades 3 to 5, which are not appropriate for F&V cultivation, are of lower quality and are mainly used to grow arable crops, such as cereals, grass and other pasture for animal grazing. In this model, we used an estimate of Grade 1–2 land (% total agricultural land).

F&V purchases were obtained from the Family Food module of the Living Costs and Food Survey (LCFS),^16^ which is an annual representative survey of UK households that collects information on food expenditure and purchasing using 2-week diaries. Data on F&V household waste were obtained from the Waste & Resources Action Programme (WRAP).^17^ WRAP used a combination of waste composition data to estimate waste collected by local authorities, composted at home, fed to animals and disposed of down the drain for different food groups, including F&V. We used data from 2012 due to the high granularity in food groups examined that year. National F&V waste was translated into F&V waste per capita measured as a percentage of F&V purchases, using information on UK population size from the Office for National Statistics (ONS) and F&V purchases from the LCFS 2012. Finally, we used F&V intake data for three age groups (25 to 44, 45 to 64, 65+), sex and IMD quintiles in England from the National Diet and Nutrition Survey Rolling Programme (NDNS) Years 1 to 4 and 7 to 8. Years 5 to 6 were not employed due to lack of data on IMD. The IMD is a relative measure of deprivation that assigns a deprivation score to small areas in England based on different socioeconomic criteria. The NDNS uses 4-day food diaries to estimate dietary intake in a nationally representative population sample in the UK. The definition of F&V across the different data sources is described in online supplementary appendix table A1.

We used projections of CHD and stroke mortality, stratified by age (25 to 34 until 85+ years), sex and quintiles of IMD using a Bayesian Age-Period-Cohort model (BAPC). The methodology of the BAPC is described in more detail elsewhere^18^ and in online supplementary appendix 1. We used relative risks (RRs) between F&V intake and CVD from meta-analyses of longitudinal studies.^19^ Specifically, RRs for CHD, ischaemic stroke and haemorrhagic stroke by serving of fruit intake and vegetable intake were used (see online supplementary appendix table A2). These RRs were chosen because they are age-specific, being adjusted for effect-modification by age, although they were assumed to not vary by sex or IMD quintiles.

### Modelled scenarios

We modelled two potential scenarios of agricultural land change. Agricultural land suitable for F&V (Grade 1–2 land) accounts for approximately 19% of total agricultural land in England, based on ALC data. However, only 1.4% of agricultural land on average was used to grow F&V in England between 2010 to 2018 with almost no variation across years (see online supplementary appendix table A3). The modelled scenarios assumed that land allocated to F&V in England would gradually increase in equal annual increments throughout the agricultural transition period, defined by the Agriculture Bill as the period between 2021 to 2027. The assumed changes in agricultural land used for F&V production under the two modelled scenarios is shown in table 2. These are plausible in terms of historical data.^11^ Overall, the scenarios assumed that by the end of the agricultural transition period (2027), land allocated to F&V would reach:

**Table 2:** Changes in fruit and vegetable land in the first year of the modelling period (2021), at the end of the agricultural transition period (2027) and at the end of the modelling period (2030) under each modelled scenario

	F&V land (% Grade 1–2 land)			F&V land (% of total agricultural land)		
	2021	2027	2030	2021	2027	2030
Baseline scenario	–	–	–	^14^	^14^	^14^
Scenario 1	–	10	10	^15^	^19^	^19^
Scenario 2	–	20	20	^18^	^39^	^39^

–, represents unknown numbers that are not needed to investigate modelled scenarios.

F&V, fruits and vegetables.

Scenario 1: 10% of Grade 1–2 land (land suitable for F&V production) in England.

Scenario 2: 20% of Grade 1–2 land (land suitable for F&V production) in England.

We translated increases in agricultural land into increases in F&V production, assuming that F&V yield and the relative difference between F&V agricultural land would not change over time. Extra agricultural production of F&V was then translated into extra F&V purchases using a purchases-to-supply ratio and assuming that all extra production would be mirrored by increased consumer demand. The ratio, estimated using data on F&V purchases and supply in the UK, adjusted for potential losses between the farm and the consumer, including losses at the packaging, distribution and retailing stages, as well as F&V used towards production of processed foods. In a one-way sensitivity analysis, we further investigated the impact of this ratio by assuming that only 50% of F&V production would be translated into F&V purchases, based on estimates on F&V production losses from the Food and Agriculture Organization.^20^ We observed no persistent trend of the ratio over time, although annual variations were taken into account in a probabilistic sensitivity analysis. We also assumed a time lag between the decision to shift agricultural land towards F&V and F&V reaching consumer baskets. This was set at 2.5 years on average for all F&V and was allowed to vary between 0 and 5 years in a probabilistic sensitivity analysis. This reflected variation in the production cycle of different crops, with some entering the market at the same year of their production, whereas others, such as apple orchards, taking at least 5 years to go through a complete production cycle.^21^

Finally, changes in F&V purchases were translated into changes in F&V intake after accounting for F&V waste at household level. Changes in F&V intake were estimated at national level and translated into changes per person per day using population projections between 2021 to 2030 from the ONS.^22^ We assumed that extra F&V intake will be distributed equally among all age, sex and IMD groups. Relevant model inputs are presented in online supplementary appendix table A3.

### The IMPACT Food Policy model

The IMPACT Food Policy model has been previously used to estimate impacts of food policies on CVD outcomes through changes in dietary intake.^9 10^ The estimated changes in dietary intake of F&V under the two modelled scenarios were translated into changes in CHD, ischaemic stroke and haemorrhagic stroke mortality, measured in Deaths Prevented or Postponed (DPPs), using mortality projections and appropriate RRs (see online supplementary appendix 2). The combined impact of changes in F&V intake was estimated using a cumulative risk-reduction approach (see online supplementary appendix 2). The cumulative number of CVD DPPs under each modelled scenario was estimated as the sum of CHD, ischaemic stroke and haemorrhagic stroke DPPs, between 2021 to 2030.

### Deterministic sensitivity analysis

We performed a sensitivity analysis to investigate the potential impact of modelled scenarios under a No Deal Brexit. In the main analysis, we assumed that Brexit would not substantially change F&V trade in England. However, depending on the agreed deal, Brexit might drastically change the UK’s trade regime with potential implications on F&V intake. A previous analysis has estimated that post-Brexit trade policy might reduce F&V intake, with the worst-case scenario being a No Deal Brexit, reducing fruit intake by −11.4% (−14.2% to −9.5%) and vegetable intake by −9.1% (−11.0% to −7.8%) in 2021.^23^ In a sensitivity analysis, we estimated the potential impact of modelled scenarios on CVD mortality between 2021 to 2030, after assuming a reduction in F&V intake in 2021 due to a No Deal Brexit.

### Probabilistic sensitivity analysis

We tested the impact of uncertainty of modelled input parameters on modelled outcomes using Monte Carlo simulation. Statistical distributions were assigned to different model inputs (see online supplementary appendix table A4). Then, the model was run across multiple iterations, using random values of model inputs derived from their respective statistical distributions. The median and 2.5^th^ and 97.5^th^ percentiles of 1000 iterations were used to estimate model outputs and their 95% uncertainty intervals (95% UI).

## Results

### Impact of modelled scenarios on F&V production

We estimated that F&V production would increase every year between 2021 to 2030, under both scenarios. Increasing F&V land until it reaches 10% of Grade 1–2 land would contribute approximately 1.1 (95% UI: 0.5 to 2.1) million tonnes of extra fruit and 4.0 (2.0 to 7.2) million tonnes of extra vegetable production between 2021 to 2030 (table 3). Under the more ambitious scenario of F&V land reaching 20% of Grade 1–2 land, we estimated that F&V production would increase by approximately 5.2 (2.9 to 8.4) million and 19.2 (11.6 to 29.2) million tonnes, respectively, between 2021 to 2030 (table 3).

**Table 3:** Estimated impact of modelled scenarios on production of fruits and vegetables in the beginning, the end and throughout the modelling period

Scenario	Change in production in thousand tonnes (95% UI)	
	Fruits	Vegetables
2021		
Scenario 1	31 (15 to 45)	115 (59 to 181)
Scenario 2	145 (97 to 190)	535 (419 to 723)
2030		
Scenario 1	222 (108 to 368)	803 (452 to 1261)
Scenario 2	1037 (677 to 1457)	3823 (2773 to 4997)
2021–2030		
Scenario 1	1097 (497 to 2062)	4037 (2003 to 7237)
Scenario 2	5177 (2946 to 8435)	19 1888 (11 576 to 29 204)

95% UI, 95% uncertainty interval.

### Impact of modelled scenarios on F&V intake

**Figure 1:**
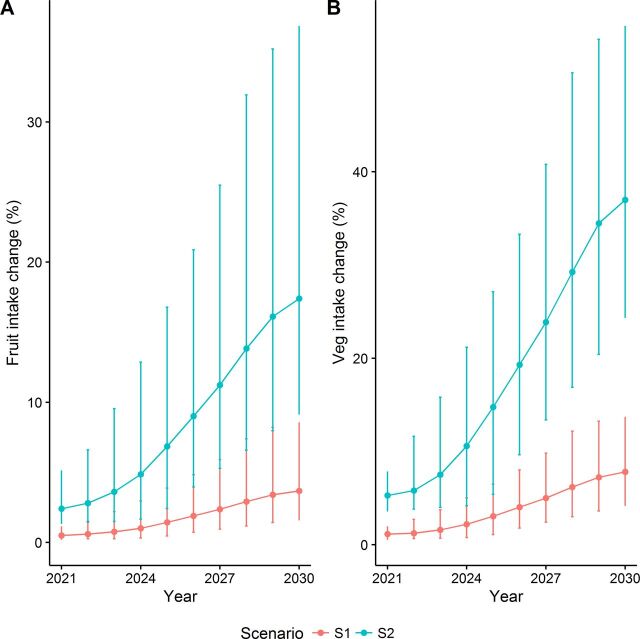
Estimated impact of modelled scenarios on intake of (A) fruits and (B) vegetables, over the modelling period.

At baseline, dietary intake among English adults aged 25 years and above was approximately 111 (SE: 3) g/day for fruits and 199 (SE: 5) g/day for vegetables, with F&V intake reducing with increasing deprivation (see online supplementary appendix table A5). We estimated that, between 2021 to 2030, F&V intake would gradually increase under both modelled scenarios (figure 1). Increasing land allocated to F&V until it reached 10% of Grade 1–2 land could increase F&V intake by approximately 3.7% (1.6% to 8.6%) and 7.8% (4.2% to 13.7%), respectively, in 2030. Similarly, if land allocated to F&V reaches 20% of Grade 1–2 land compared with maintaining current allocations, we estimated that F&V intake would be approximately 17.4% (9.1% to 36.9%) and 37% (24.3% to 55.7%) higher by 2030 (see online supplementary appendix table A6).

### Impact of modelled scenarios on CVD mortality

We projected that, between 2021 to 2030, CHD and stroke would generate approximately 435 200 (330 500 to 584 500) and 258 500 (148 400 to 532 500) cumulative deaths, respectively, in England, under a baseline business-as-usual scenario. Estimated CHD deaths were increasing with deprivation, with approximately 23% of CHD deaths in 2030 in the most deprived group compared with 15% in the least deprived group. Number of stroke deaths was projected to be more equitable, with smaller differences across IMD quintiles.

We estimated that increasing land allocated to F&V until it reaches 10% of Grade 1–2 land was associated with approximately 1230 (630 to 2150) fewer CHD and 2660 (1320 to 4930) fewer stroke deaths between 2021 to 2030. Under the scenario that land allocated to F&V would reach 20% of Grade 1–2 land, we estimated 5750 (3250 to 8910) fewer CHD and 12 260 (6590 to 19 960) fewer stroke deaths between 2021 to 2030 (see online supplementary appendix table A7). In 2030, the two scenarios reduced CVD mortality by 1.3% and 6.0%, respectively (see online supplementary appendix table A8).

### Impact of modelled scenarios on inequalities

**Figure 2:**
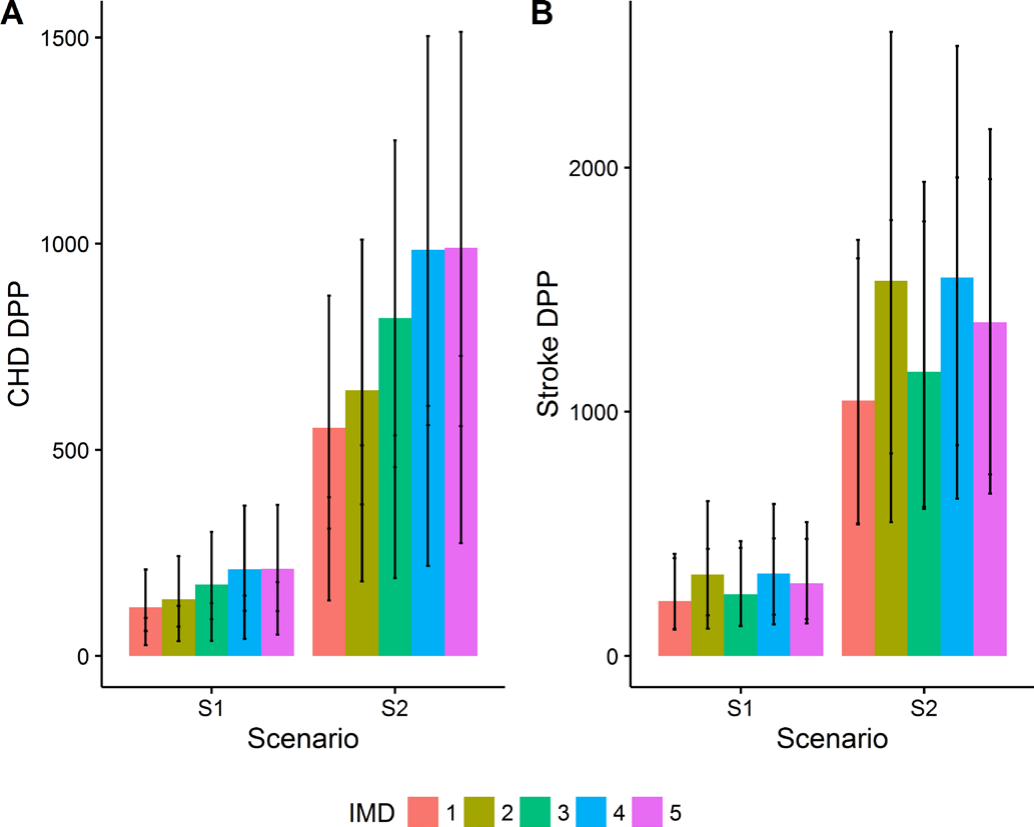
Estimated impact of modelled scenarios on (A) cumulative coronary heart disease mortality and (B) stroke mortality, by Index of Multiple Deprivation (IMD), 2021 to 2030. CHD, coronary heart disease; DPP, Deaths Prevented or Postponed. IMD 5 is the most deprived group

Across both scenarios, F&V intake was estimated to increase more in the most deprived groups compared with the least deprived (see online supplementary appendix table A6). We estimated that the most deprived group would yield the highest number of CHD DPPs, under both scenarios (figure 2). For example, increasing land allocated to F&V until it reaches 20% of Grade 1–2 land could save approximately 1470 (830 to 2240) CHD deaths in the most deprived group compared with 790 (440 to 1260) in the least deprived, between 2021 to 2030 (see online supplementary appendix table A7). We also estimated that the second and fourth most deprived quintile groups would be the most benefited in terms of stroke outcomes under both scenarios (figure 2). The smallest impact occurred in the least deprived group, which accounted for 17% of stroke DPPs, whereas 43% of stroke DPPs occurred in the two most deprived groups combined.

### Sensitivity analyses

**Figure 3:**
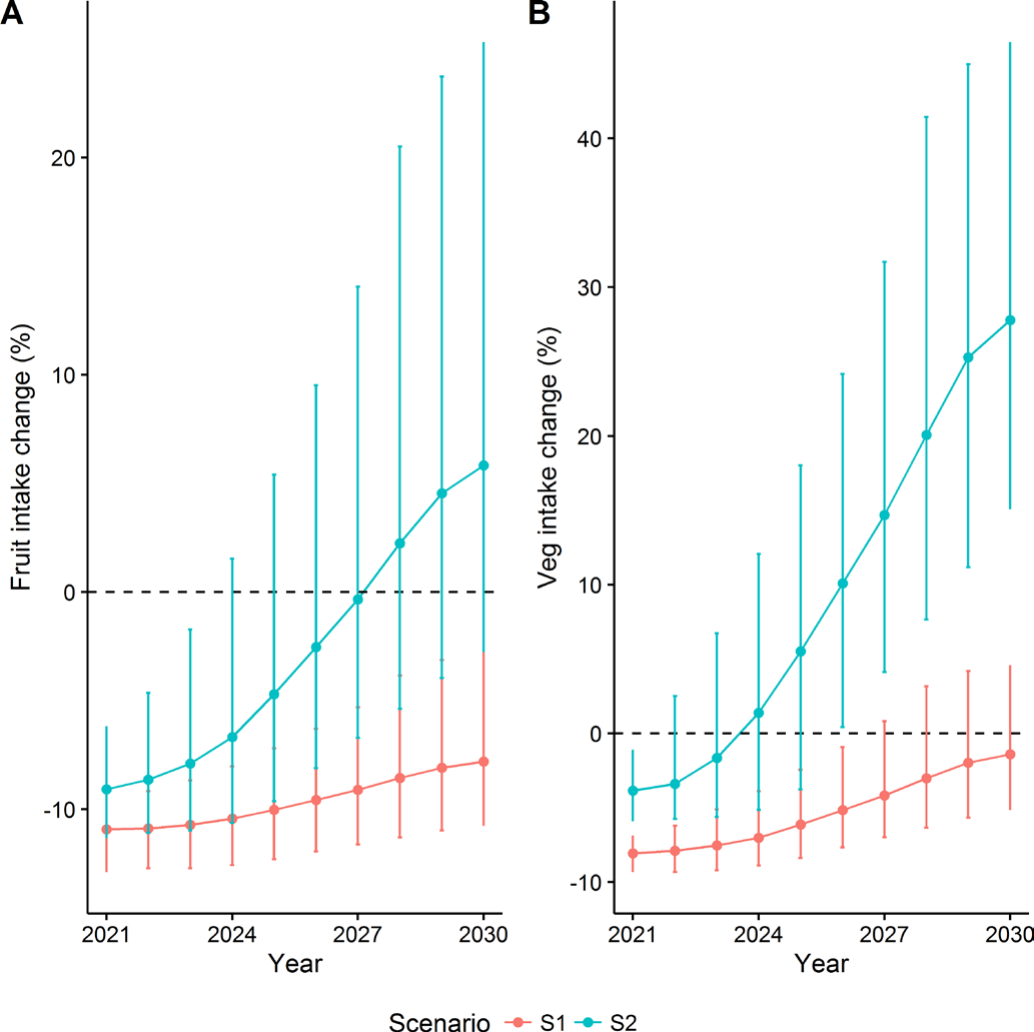
Estimated impact of modelled scenarios on intake of (A) fruits and (B) vegetables, under a No Deal Brexit, over the modelling period. Results from sensitivity analysis.

In a sensitivity analysis, we considered the potential impact of a No Deal Brexit on F&V intake in 2021. We estimated that the impact of increasing land allocated to F&V until it reaches 10% of Grade 1–2 land would fail to offset the negative impacts of a No Deal Brexit on F&V intake (figure 3), increasing CVD mortality by approximately 6900 (4290 to 10 190) additional deaths between 2021 to 2030 (table 4). In contrast, increasing F&V land until it reaches 20% of Grade 1–2 land would increase intake of F&V even in a case of a No Deal Brexit (see online supplementary appendix table A9), preventing or postponing approximately 7300 (1520 to 16 080) CVD deaths between 2021 to 2030. These would mostly occur in the most deprived group, which accounted for 28% of CVD DPPs, compared with 13% CVD DPPs in the least deprived group. In a second sensitivity analysis, assuming only 50% of F&V production to be translated into F&V purchases would still largely benefit CVD outcomes in England under both scenarios, although effects were moderately lower (see online supplementary appendix tables A10–11).

**Table 4:** Estimated impact of modelled scenarios on cumulative coronary heart disease, stroke and cardiovascular disease mortality, stratified by Index of Multiple Deprivation (IMD), under a No Deal Brexit, 2021 to 2030. Results from sensitivity analysis

Scenario	Coronary heart disease	Stroke	Cardiovascular disease
Scenario 1			
First IMD quintile	−390 (−580 to –260)	−930 (−1350 to –610)	−1330 (−1920 to –870)
Second IMD quintile	−490 (−710 to –330)	−1150 (−1650 to –740)	−1640 (−2360 to –1070)
Third IMD quintile	−550 (−810 to –370)	−970 (−1430 to –620)	−1520 (−2240 to –980)
Fourth IMD quintile	−460 (−690 to –290)	−850 (−1270 to –490)	−1310 (−1960 to –780)
Fifth IMD quintile	−430 (−650 to –250)	−670 (−1060 to –340)	−1100 (−1710 to –580)
*Total*	*−2330 (−3430 to –1500*)	*−4570 (–6760 to –2780*)	*−6900 (–10 190 to –4290*)
Scenario 2			
First IMD quintile	220 (–10 to 580)	700 (50 to 1710)	920 (40 to 2280)
Second IMD quintile	260 (–30 to 680)	940 (80 to 2290)	1200 (60 to 2970)
Third IMD quintile	330 (–10 to 850)	830 (110 to 1980)	1160 (110 to 2830)
Fourth IMD quintile	610 (170 to 1230)	1340 (420 to 2750)	1950 (590 to 3980)
Fifth IMD quintile	710 (240 to 1360)	1360 (490 to 2660)	2070 (730 to 4020)
*Total*	*2130 (360 to 4710*)	*5160 (1160 to 11 380*)	*7300 (1520 to 16 080*)

The fifth IMD quintile is the most deprived

## Discussion

This, to our knowledge, is the first study to estimate the potential effects on health and health inequalities via changes in F&V intake of proposed changes in the Agriculture Bill in post-Brexit Britain. We found that the post-Brexit agricultural policy has the potential to increase intake of F&V and reduce health inequalities by increasing agricultural land allocated to F&V in England. We estimated that gradually increasing F&V land throughout the agricultural transition period (2021 to 2027) until it reaches 10% and 20% of land suitable for F&V production (Grade 1–2 land) could prevent or postpone approximately 3890 CVD deaths (0.6% lower CVD mortality) and 18 010 CVD deaths (2.6% lower CVD mortality), respectively, between 2021 to 2030, when compared with a baseline scenario. Importantly, we found that under both modelled scenarios, 45% of deaths averted would occur in the two most deprived groups, thus reducing CVD inequalities.

This study builds substantially on previous work in this field, which is however largely focussed on estimating the impact of changes in diet on land use. A modelling study suggested that following the Eatwell Plate guidelines in the UK would require an increase in UK and non-UK horticultural land of 10 000 and 20 000 hectares, respectively.^24^ Similarly, complying with UK dietary guidelines, including an increase in F&V consumption to reach 400 g/day, was estimated to increase land use in England and Wales by 47.7% for vegetables, 55.4% for fruit grown on trees and 103.5% for fruit grown on bushes.^25^ Our study found that gradually increasing F&V land throughout the agricultural transition period by approximately 36% (from 1.4% to 1.9% of total agricultural land) or 179% (from 1.4% to 3.9% of total agricultural land) would increase F&V intake by approximately 20 g/day or 86 g/day, respectively, in 2030. While these increases would produce beneficial improvements for CVD outcomes, average F&V consumption would remain below recommended levels of 400 g/day. Our modelled increases in F&V land is comparable with historical data, which show that horticultural land in England between 1983 to 1990 accounted for 1.9% of total agricultural land on average, not exceeding the modelled increases under our first, least ambitious scenario.^11^ Thus, we believe that the potential changes we have modelled are plausible.

The current proposal for the post-Brexit agricultural policy is focussing on the protection and improvement of the environment. However, without explicitly having the additional aim of improving the public’s health through addressing poor diet represents a significant missed opportunity. Incorporating supply-side interventions that promote F&V production in the Agriculture Bill can improve CVD outcomes and reduce associated inequalities, highlighting England’s capacity to support healthy eating through British-grown F&V. F&V production can be promoted through various strategies, including agricultural subsidies linked to F&V production, improving access to F&V land, support horticulture seasonal workers and invest in relevant research and development. Increasing F&V production, however, is clearly just one part of the multifaceted approach required to radically tackle the growing diet-related burden of ill-health. Our analysis assumed that all extra F&V production would be met by consumer demand. To achieve this, the post-Brexit agricultural policy should be part of an integrated ‘farm-to-fork’ food strategy that promotes the healthy choice as the easy, accessible and affordable choice across the supply chain. The recently launched UK Food Strategy has the potential to do this. However, this will require sustained leadership and commitment to achieve an agricultural policy that aligns a sustainable food system with public health goals.

This study is topical, offering a timely investigation of the potential post-Brexit agricultural policy at the time of its development, using a previously validated model^9 10^ and high quality data inputs. Model scenarios have been informed using data on ALC in England, taking into account the capacity of agricultural land to increase F&V production, while the scope of the post-Brexit agricultural policy to offset the potential negative implications of a No Deal Brexit on F&V intake was investigated in a sensitivity analysis. However, this study also has some limitations. Data inputs were not always available for England, thus they were approximated using UK data (table 1). Scenarios were informed using the only available national data on ALC, which were developed using information on land quality between 1967 to 1974. Although these cannot be used for current evaluation of agricultural land of specific sites, they are appropriate for general agricultural planning at national level.^26^ Moreover, the model used an estimate for F&V waste that does not incorporate F&V disposed as part of cooked dishes or out-of-home, while it does not correct for potential changes in F&V weight due to cooking.^17^ However, waste estimates include a wide range of waste disposal channels at household level and provide information on disaggregated food groups. The model also assumed that no changes would occur in model inputs throughout the modelling period. Wherever possible, mean estimates of data from the latest available years were used to consider annual variations. In this model, the change in agricultural supply was translated into change in purchases using a purchases-to-supply ratio, which might not accurately represent losses between the farm and the consumer. We further tested the strength of this estimate through a sensitivity analysis that assumed only half of F&V supply reaching consumer baskets, which provided comparable results. As the mechanism through which changes in production would increase intake is not specified in our analysis, changes in prices or purchasing power post-Brexit were not explicitly modelled. This analysis did not investigate potential changes in consumer behaviour due to different preferences in imported and British-grown F&V. Finally, this model relied on some necessary assumptions (see online supplementary appendix table A12).

Post-Brexit agricultural policy presents an opportunity to improve dietary intake in the UK, with beneficial impacts on CVD mortality and inequalities. However, this is not inevitable and the production of British-grown F&V as part of a comprehensive agricultural strategy that intervenes across the whole supply chain must be supported. A failure to integrate public health aims in the new UK agricultural policy would mean a missed opportunity to be a global leader in orientating the food system towards health and planetary goals.
