# Supplementary material for: Potential impacts of post-Brexit agricultural policy on fruit and vegetable intake and cardiovascular disease in England: a modelling study
Source: BMJ Nutr Prev Health. 2020 Jan 14;3(1):3–10. doi: 10.1136/bmjnph-2019-000057 (PMC7664506; doi:10.1136/bmjnph-2019-000057)
Supplement: Supplementary data [file bmjnph-2019-000057supp001.pdf]

Supplementary Appendix

Supplementary Appendix 1: Cardiovascular mortality projections

We used a Bayesian Age-Period-Cohort (BAPC) model to estimate CHD and stroke mortality projections between 2021 and 2030, by age, sex, and IMD. The BAPC model assumes that historic changes in mortality due to population's age of death, calendar period of death, and cohort of birth will continue in the future. We fitted the model using the BAMP (Bayesian Age-Period-Cohort Modeling and Prediction) software, which employs Markov Chain Monte Carlo simulations for mortality predictions and their 95% Credible Intervals. We used historic population and CHD and stroke mortality data between 1981 and 2016 from the ONS, and ONS population projections between 2017 and 2030. As the ONS does not provide population estimates and projections by IMD for all years used in this model, we assumed that the relative differences in population estimates across IMD quintiles by age and sex group between 1981 and 2030 were equal to the relative differences in 2015. CHD and stroke were defined using ICD-9 and ICD-10 codes as described in Table 1.1. Overall stroke projections were further adjusted to represent ischaemic and haemorrhagic stroke projections, using an ischaemic-to-haemorrhagic stroke mortality ratio from 2016 (Table 1.2) and assuming that no changes will occur in stroke clinical care.

Table 1.1. ICD codes for CVD outcomes

Type of CVD outcomes	ICD-9 codes (1981-2000)	ICD-10 codes (2001-2016)
Coronary heart disease	410-414	I20-I25
Overall stroke	430-438	I60-I69
Ischaemic stroke		I63, I65-I67 (except I67.4)
Haemorrhagic stroke		I60-I62, I69.0-I69.2, I67.4
Other (not specified) stroke		I64, I69.4, I69.8

ICD, International Statistical Classification of Diseases and Related Health Problems; CVD, cardiovascular disease

Seferidi P, *et al. bmjnph* 2020; 3:e000057. doi: 10.1136/bmjnph-2019-000057

Table 1.2. Percentage of stroke deaths attributed to ischaemic, haemorrhagic, and other (not specified) stroke and ischaemic-to-haemorrhagic stroke ratio

**Population group**	**Ischaemic stroke**	**Haemorrhagic stroke**	**Other (not specified) stroke**	**Ischaemic-to-haemorrhagic stroke ratio***
Men 25-34	14%	82%	5%	0.22
Men 35-44	12%	79%	9%	0.26
Men 45-54	17%	67%	16%	0.50
Men 55-64	18%	53%	30%	0.90
Men 65-74	23%	37%	40%	1.71
Men 75-84	24%	28%	48%	2.57
Men 85+	30%	19%	51%	4.25
Women 25-34	23%	69%	9%	0.46
Women 35-44	14%	81%	5%	0.23
Women 45-54	14%	73%	13%	0.38
Women 55-64	15%	63%	23%	0.59
Women 65-74	18%	46%	36%	1.16
Women 75-84	23%	32%	46%	2.13
Women 85+	30%	17%	53%	4.98
Overall	*26%*	*28%*	*46%*	*2.58*

*Other (not specified) stroke was combined with ischaemic stroke.

*Estimated using ONS mortality data from 2016

Seferidi P, *et al. bmjnph* 2020; 3:e000057. doi: 10.1136/bmjnph-2019-000057

Supplementary Appendix 2: Deaths prevented or postponed calculations

We used the IMPACT Food Policy model to translate changes in F&V intake into deaths prevented or postponed (DPPs) for each age, sex, and IMD group, every year between 2021 and 2030. DPPs are estimated as shown in the equation below:

$$DPPs = \left(1 - e^{beta \times IntakeChange}\right) * Mortality,$$

Where beta is the natural logarithm of the relative risk between fruit or vegetable intake and coronary heart disease, ischaemic stroke or haemorrhagic stroke, IntakeChange is the estimated change in fruit or vegetable intake under each modelled scenario, and Mortality is the projected number of deaths under a baseline business-as-usual scenario. Where relevant, parameters of this equation were specific for each age, sex, IMD group, and year of the modelling period.

Fruit intake and vegetable intake are independently associated with CVD outcomes. At the same time, fruit intake and vegetable intake might be correlated due to common drivers of dietary behaviour. Thus, we expressed the combined impact of fruit intake and vegetable intake on CVD using a cumulative risk-reduction approach, as previously implemented by Bajekal et al[1]. First, we estimated an adjustment factor, as shown in the equation below:

$$AF = CR/AR$$

where

$$CR = 1 - \left(1 - abs\left(MortalityChange_{fruit}\right)\right) * \left(1 - abs\left(MortalityChange_{\mathrm{veg}}\right)\right)$$

and

$$AR = abs\left(MortalityChange_{fruit}\right) + abs\left(MortalityChange_{\mathrm{veg}}\right)$$

with $\mathrm{MortalityChange_{fruit}}$ and $\mathrm{MortalityChange_{veg}}$ being the change in mortality change attributed to fruit and vegetable intake respectively and estimated as $\left(1 - e^{beta \times IntakeChange}\right)$ similar to the equation above.

The estimated of overall DPPs were then estimated

$$DPPs_{overall} = \left(DPPs_{fruit} + DPPs_{veg}\right) * AF$$

where $\mathrm{DPPs_{fruit}}$ and $\mathrm{DPPs_{veg}}$ being the DPPs attributed to changes in fruit and vegetable intake, respectively.

Seferidi P, *et al. bmjnph* 2020; 3:e000057. doi: 10.1136/bmjnph-2019-000057

Supplementary Tables

Table A1. Classification of fruit and vegetables across different data sources

Data Source	Fruit	Vegetables
June Survey of Agriculture (land data)	Orchards, Small fruit, Area under glass or plastic covered structure used for ‘vegetables, salad, and fruit’*	Vegetables for human consumption (Excludes potatoes, peas for harvesting dry, which are mainly used for stock feeding, and mushrooms), Area under glass or plastic covered structure used for ‘vegetables, salad, and fruit’* and mushrooms
Horticulture Statistics (production data)	Total fruit (includes Orchard fruit and Soft fruit)	Field vegetables (includes Roots and Onions, Brassicas, Legumes, Others) Protected vegetables
Horticulture Statistics (import and export data)	Total fruit (includes Orchard fruit and Soft fruit). Exports includes re-exported fruit	Total vegetables, excluding potatoes and sweetcorn
Waste and Resources Action Programme (waste data)	Banana; Orange; Apple; Melon; Pineapple; Other citrus; Stone fruit; Soft / berry fruit; Pear; All other fresh fruit	Carrot; Onion; Other root vegetables; Cabbage; Lettuce; Cauliflower; Tomato; Broccoli; Cucumber; Pepper; Mixed vegetables; Leafy salad; Mushroom; Leek; Bean (all varieties); Spring onion; All other fresh vegetables and salads
Living Costs and Food Survey 2016/7 (purchase data)	Home purchases (includes Fresh fruit; Frozen strawberries, apple slices, peach halves, oranges and other frozen fruits)<br>Eating out (includes Fresh fruit)	Home purchases (includes Fresh green vegetables; Other fresh vegetables; Peas, frozen; Beans, frozen; Other frozen vegetables)<br>Eating out (includes Green vegetables; Other fresh vegetables, excluding peas and sweetcorn and baked beans; Root vegetables; Mushrooms; Mixed vegetables or unspecified 'vegetable'; Other vegetables; Green salads without dressing)
National Diet and Nutrition Survey Rolling Programme, Years 1-4 & 7-8 (intake data)	Fruit, including fresh and dried fruit and smoothies but not including juice	Vegetables, including legumes

Where possible, starchy vegetables such as potatoes and corn were excluded to stay consistent with the definition of fruits and vegetables in Micha et al, 2017[1] that provided the relative risks used in the model.
*50% of Area under glass or plastic covered structure used for ‘vegetables, salad, and fruit’ was allocated to

fruits and 50% to vegetables, as more granular data were not available. This area covers approximately 0.5% of total horticultural land. Types of crops in glasshouse area in England were estimated as their mean between 2015 and 2017[2].

Table A2. Relative risks for CHD, ischaemic stroke, and haemorrhagic stroke per serving of fruit or vegetable consumption

	**RR per serving of fruit consumption**			**RR per serving of vegetable consumption**		
Age group	CHD	Ischaemic stroke	Haemorrhagic stroke	CHD	Ischaemic stroke	Haemorrhagic stroke
25-34	0.92 (0.87, 0.97)	0.83 (0.76, 0.9)	0.63 (0.49, 0.81)	0.93 (0.89, 0.97)	0.76 (0.64, 0.9)	0.76 (0.61, 0.95)
35-44	0.92 (0.87, 0.97)	0.83 (0.77, 0.9)	0.64 (0.5, 0.82)	0.93 (0.9, 0.97)	0.77 (0.66, 0.9)	0.77 (0.62, 0.95)
45-54	0.93 (0.89, 0.97)	0.86 (0.8, 0.92)	0.69 (0.56, 0.84)	0.94 (0.91, 0.97)	0.80 (0.7, 0.92)	0.80 (0.67, 0.96)
55-64	0.94 (0.91, 0.98)	0.88 (0.83, 0.93)	0.73 (0.61, 0.87)	0.95 (0.93, 0.98)	0.83 (0.74, 0.93)	0.83 (0.72, 0.96)
65-74	0.95 (0.92, 0.98)	0.90 (0.86, 0.94)	0.77 (0.67, 0.89)	0.96 (0.94, 0.98)	0.86 (0.78, 0.94)	0.86 (0.76, 0.97)
75+	0.97 (0.96, 0.99)	0.94 (0.92, 0.96)	0.86 (0.8, 0.92)	0.98 (0.97, 0.99)	0.92 (0.87, 0.96)	0.92 (0.86, 0.97)

One serving of fruits or vegetables equals to 100 g per day.
CHD, coronary heart disease; RR, relative risk
Source: Micha et al, 2017[3]

Seferidi P, *et al. bmjnph* 2020; 3:e000057. doi: 10.1136/bmjnph-2019-000057

Table A3. Fruit and vegetable related inputs of the model

	**Fruit**	**Vegetables**
Land (% total agricultural land) (mean 2010-2018, SD)	0.3% (0.0%)	1.1% (0.1%)
Land (% total fruit and vegetable agricultural land) (mean 2010-2018, SD)	24% (1.4%)	76% (1.4%)
Yield (tonnes/hectare) (mean 2010-2018, SD)	19.5 (2.8)	22.0 (1.2)
Supply (thousand tonnes) (mean 2008-2017, SD)*	4,166 (417)	4,599 (152)
Purchases at home and eating out (thousand tonnes per year) (mean 2008-2016/7, SD)*	2,612 (102)	2,820 (84)
Household waste 2012 (g/p/w) (point estimate, 95% CI)**	274 (238, 311)	268 (230, 305)
Purchases at home 2012 (g/p/w)**	744	734

*inputs used to estimate purchases-to-supply ratio
**inputs used to estimate F&V waste as a percentage of fruit and vegetable purchases
SD, standard deviation; 95% CI, 95% confidence interval; g/p/w, grams per person per week

Seferidi P, *et al. bmjnph* 2020; 3:e000057. doi: 10.1136/bmjnph-2019-000057

Table A4. Statistical distributions and parameters for model inputs used in the probabilistic sensitivity analysis

**Inputs**	**Distribution**	**Parameters**	**Source**
F&V land	Normal	Mean and SD of 2010-2018 average land	DEFRA[4]
F&V yield	Normal	Mean and SD of 2010-2018 average yield	DEFRA[5]
F&V supply	Normal	Mean and SD of 2010-2018 average supply	DEFRA[6]
F&V purchases	Normal	Mean and SD of 2008-2016/7 average purchases	LCFS[7]
Waste at household level	Normal	Mean: F&V waste estimates in 2012<br>SD: estimated from 95% CI of F&V waste estimates	WRAP[8]
F&V consumption	Normal	Mean: mean consumption by age, sex, and IMD<br>SD: SE of the mean	NDNS RP Years 1-4 & 7-8[9]
RR for CHD/ischaemic stroke/haemorrhagic stroke per fruit or vegetable serving	Log normal	RR and SE(lnRR) estimated from 95% CI, by age	Micha, 2017[3] Parameters based on Barendregt, 2010[10]
CHD/ischaemic stroke/haemorrhagic stroke deaths, 2021-2030	Pert	mode: death projections, best estimate<br>min: death projections, lower 95% confidence limit<br>max: death projections, upper 95% confidence limit<br>by age, sex, and IMD	Own estimations
Effect of no deal Brexit on F&V consumption by IMD	Pert	mode: best estimate; min: lower 95% uncertainty limit; max: upper 95% uncertainty limit	Seferidi, 2019[11]

F&V, fruit and vegetables; SD, standard deviation; DEFRA, Department for Environment, Food & Rural Affairs; LCFS, Living Costs and Food Survey; WRAP, Waste & Resources Action Programme; NDNS RP, National Diet and Nutrition Survey Rolling Programme; IMD, Index of Multiple Deprivation; RR, relative risk; SE, standard error, CHD, coronary heart disease; 95% CI, 95% confidence interval

Table A5. Baseline intake of fruits and vegetables in England by age, sex, and IMD. Means (grams/day) and standard errors and % of the overall sample meeting the 5-a-day targets.

**Age/Sex group**	**Overall**	**IMD 1**	**IMD 2**	**IMD 3**	**IMD 4**	**IMD 5**
Fruit						
Men 25-44	86.8 (7.6)	111 (33.5)	88.2 (9.8)	80.9 (12.2)	70.1 (11.5)	84.3 (10.2)
Men 45-64	120.1 (6.5)	119.6 (9)	144.8 (15.1)	120.1 (13.1)	109.5 (15)	106.9 (22.8)
Men 65+	117.1 (7.5)	136.6 (15.1)	137.7 (17)	138.6 (24.7)	92.9 (12.4)	76.6 (12.7)
Women 25-44	93.4 (4.5)	119.6 (12.3)	91.3 (8)	95.1 (10.8)	79.8 (7.5)	85.8 (10.7)
Women 45-64	127.1 (5)	146.3 (11.2)	146.3 (10.9)	122.8 (11.9)	107.2 (10.2)	109.2 (11.1)
Women 65+	128.7 (7.7)	154.2 (13.5)	149.2 (18.8)	121.2 (17.2)	114.8 (17.2)	67.2 (15.6)
Total	110.6 (2.7)	*132.2 (6.7)*	*123.8 (5.4)*	*109 (5.7)*	*93.9 (4.9)*	*91.1 (6.3)*
Vegetables						
Men 25-44	193.6 (6.9)	223.1 (23.9)	190.5 (10.8)	204.2 (13.3)	165.8 (14.9)	184.6 (11.9)
Men 45-64	200.2 (5.9)	228.8 (13.2)	204.7 (10.2)	197.1 (15.8)	179.8 (13.6)	180.3 (12.2)
Men 65+	188.7 (7.8)	167.5 (11.8)	202.6 (15.7)	232.7 (25.3)	171 (13.3)	174.7 (19.3)
Women 25-44	188.5 (5.3)	194.8 (11.3)	180 (8.9)	208.8 (15.6)	181.2 (9)	179 (12)
Women 45-64	199.1 (5.1)	210.1 (7.8)	217.2 (13.3)	207.9 (13)	170.2 (11.3)	183.4 (12.9)
Women 65+	173.9 (5.4)	201.5 (9.9)	174.2 (10.2)	158.2 (9.4)	155.6 (11.4)	147.1 (16.6)
Total	191.7 (2.7)	*206.7 (5.8)*	*195.4 (5)*	*199.9 (6.6)*	*172.6 (5.2)*	*178.2 (5.7)*
Meeting 5-a-day targets	32%	38%	37%	31%	24%	25%

IMD 1 is the least deprived group and IMD 5 the most deprived.
IMD, Index of Multiple Deprivation
Data from the National Diet and Nutrition Survey Rolling Programme Years 1-4 and 7-8

Table A6. Estimated impact of modelled scenarios on fruit and vegetable intake overall and by IMD, in 2030

**Scenario**	**Change in consumption (95% UI)**	
	**Fruits**	**Vegetables**
Scenario 1		
1st IMD quintile	3% (1.4%, 6.3%)	7.3% (3.9%, 12.7%)
2nd IMD quintile	3.2% (1.4%, 7%)	7.5% (4.1%, 12.7%)
3rd IMD quintile	3.5% (1.6%, 7.4%)	7.3% (3.9%, 13%)
4th IMD quintile	4.2% (1.9%, 8.8%)	8.6% (4.7%, 14.4%)
5th IMD quintile	4.8% (2.1%, 10.5%)	8.4% (4.6%, 14.8%)
Total	*3.7% (1.6%, 8.6%)*	*7.8% (4.2%, 13.7%)*
Scenario 2		
1st IMD quintile	14.3% (8.3%, 27%)	34.3% (23.1%, 51.2%)
2nd IMD quintile	14.9% (8.3%, 29.8%)	35.7% (24.6%, 50.6%)
3rd IMD quintile	16.7% (9.2%, 31.8%)	34.5% (22.7%, 53.5%)
4th IMD quintile	20% (11%, 37.7%)	40.8% (28.3%, 57.8%)
5th IMD quintile	22.7% (12.1%, 45.4%)	39.8% (27.2%, 60.4%)
Total	*17.4% (9.1%, 36.9%)*	*37% (24.3%, 55.7%)*

Table A7. Estimated impact of modelled scenarios on cumulative CHD, stroke, and CVD mortality, stratified by IMD, 2021-2030

**Scenario**	**Coronary heart disease**	**Stroke**	**Cardiovascular disease**
Scenario 1			
1st IMD quintile	170 (90, 300)	440 (220, 820)	610 (300, 1120)
2nd IMD quintile	210 (110, 360)	570 (280, 1070)	770 (380, 1440)
3rd IMD quintile	240 (130, 430)	500 (250, 910)	740 (370, 1340)
4th IMD quintile	290 (150, 510)	600 (300, 1100)	890 (450, 1610)
5th IMD quintile	310 (160, 540)	560 (280, 1030)	870 (440, 1570)
Total	*1230 (630, 2150)*	*2660 (1320, 4930)*	*3890 (1950, 7080)*
Scenario 2			
1st IMD quintile	790 (440, 1260)	2060 (1080, 3330)	2850 (1520, 4590)
2nd IMD quintile	970 (550, 1520)	2610 (1380, 4340)	3570 (1930, 5860)
3rd IMD quintile	1150 (650, 1790)	2280 (1210, 3720)	3430 (1860, 5510)
4th IMD quintile	1380 (780, 2110)	2760 (1510, 4460)	4130 (2290, 6570)
5th IMD quintile	1470 (830, 2240)	2560 (1410, 4110)	4030 (2240, 6350)
Total	*5750 (3250, 8910)*	*12260 (6590, 19960)*	*18010 (9840, 28870)*

Table A8. Estimated absolute and relative impact of modelled scenarios on CHD, stroke, and CVD mortality, in 2030

		**Scenario 1**	**Scenario 2**
CVD	Deaths at baseline	65020 (35750, 141940)	65020 (35750, 141940)
	Attributable deaths	850 (1360, 500)	3900 (5430, 2640)
	Mortality increase (%)	1.3%	6.0%
CHD	Deaths at baseline	40250 (25830, 64140)	40250 (25830, 64140)
	Attributable deaths	250 (380, 160)	1170 (1570, 840)
	Mortality increase (%)	0.6%	2.9%
Stroke	Deaths at baseline	24770 (9920, 77800)	24770 (9920, 77800)
	Attributable deaths	600 (980, 340)	2720 (3860, 1790)
	Mortality increase (%)	2.4%	11.0%

Table A9. Estimated impact of modelled scenarios on fruit and vegetable intake overall and by IMD, under a no deal Brexit, in 2030. Results from sensitivity analysis.

**Scenario**	**Change in consumption (95% UI)**	
	**Fruits**	**Vegetables**
Scenario 1		
1st IMD quintile	-8.5% (-11.1%, -4.8%)	-1.9% (-5.4%, 3.6%)
2nd IMD quintile	-8.3% (-11%, -4.2%)	-1.7% (-5.2%, 3.6%)
3rd IMD quintile	-8% (-10.8%, -3.8%)	-1.9% (-5.5%, 3.9%)
4th IMD quintile	-7.3% (-10.4%, -2.4%)	-0.6% (-4.6%, 5.3%)
5th IMD quintile	-6.7% (-10.1%, -0.8%)	-0.8% (-4.7%, 5.7%)
Total	*-7.8% (-10.8%, -2.8%)*	*-1.4% (-5.2%, 4.6%)*
Scenario 2		
1st IMD quintile	2.7% (-3.6%, 15.6%)	25.2% (13.8%, 42%)
2nd IMD quintile	3.3% (-3.6%, 18.5%)	26.4% (15.3%, 41.4%)
3rd IMD quintile	5.2% (-2.7%, 20.4%)	25.3% (13.5%, 44.3%)
4th IMD quintile	8.4% (-0.8%, 26.2%)	31.6% (19%, 48.7%)
5th IMD quintile	11.1% (0.2%, 33.8%)	30.6% (17.9%, 51.2%)
Total	*5.8% (-2.8%, 25.3%)*	*27.8% (15.1%, 46.5%)*

Table A10. Estimated impact of modelled scenarios on fruit and vegetable intake overall and by IMD, 2030, with purchases-to-supply ratio=50%. Results from sensitivity analysis.

**Scenario**	**Change in consumption (95% UI)**	
	**Fruits**	**Vegetables**
Scenario 1		
1st IMD quintile	2.4% (1.1%, 5.1%)	5.9% (3.1%, 10.6%)
2nd IMD quintile	2.5% (1.1%, 5.5%)	6.1% (3.3%, 10.7%)
3rd IMD quintile	2.8% (1.2%, 6%)	5.9% (3.1%, 10.9%)
4th IMD quintile	3.3% (1.5%, 7.1%)	7% (3.8%, 12.2%)
5th IMD quintile	3.8% (1.6%, 8.4%)	6.8% (3.7%, 12.3%)
Total	*2.9% (1.2%, 6.8%)*	*6.3% (3.3%, 11.5%)*
Scenario 2		
1st IMD quintile	11.3% (6.6%, 21.6%)	27.9% (17.9%, 43.2%)
2nd IMD quintile	11.7% (6.5%, 23.4%)	29% (18.9%, 42.7%)
3rd IMD quintile	13.2% (7.2%, 25.5%)	28.1% (17.6%, 44.9%)
4th IMD quintile	15.7% (8.6%, 30%)	33.2% (21.9%, 49%)
5th IMD quintile	17.9% (9.5%, 35.5%)	32.4% (21.1%, 50.6%)
Total	*13.7% (7.1%, 29%)*	*30.1% (18.9%, 46.7%)*

Table A11. Estimated impact of modelled scenarios on cumulative CHD, stroke, and CVD mortality, stratified by IMD, with purchases-to-supply ratio=50%, 2021-2030. Results from sensitivity analysis

**Scenario**	**Coronary heart disease**	**Stroke**	**Cardiovascular disease**
Scenario 1			
1st IMD quintile	140 (70, 240)	360 (170, 680)	500 (240, 920)
2nd IMD quintile	170 (80, 300)	460 (220, 870)	620 (300, 1170)
3rd IMD quintile	200 (100, 350)	400 (190, 740)	590 (290, 1090)
4th IMD quintile	230 (120, 410)	490 (240, 890)	720 (360, 1310)
5th IMD quintile	250 (120, 450)	450 (230, 840)	700 (350, 1280)
Total	*980 (490, 1760)*	*2150 (1050, 4020)*	*3130 (1540, 5770)*
Scenario 2			
1st IMD quintile	630 (360, 1040)	1660 (890, 2840)	2290 (1240, 3880)
2nd IMD quintile	770 (430, 1260)	2110 (1120, 3600)	2880 (1550, 4850)
3rd IMD quintile	910 (520, 1480)	1830 (990, 3090)	2740 (1510, 4570)
4th IMD quintile	1100 (600, 1750)	2220 (1210, 3710)	3320 (1810, 5460)
5th IMD quintile	1170 (640, 1880)	2060 (1140, 3450)	3230 (1780, 5330)
Total	*4590 (2540, 7410)*	*9880 (5350, 16680)*	*14470 (7890, 24090)*

Seferidi P, *et al. bmjnph* 2020; 3:e000057. doi: 10.1136/bmjnph-2019-000057

Table A12. Model assumptions

**Policy scenarios**
All F&V in England are grown in Grade 1 and 2 land
Relative difference between fruit and vegetable agricultural land would not change
All extra F&V production would be used for domestic consumption
All extra F&V consumption would be equally distributed across age, sex, and IMD groups
F&V demand will increase until it meets extra supply
Labour demand to increase F&V production will be met
**Effects of F&V intake on CVD mortality**
There is an immediate effect of increasing F&V intake on CVD mortality.
There is a linear association between CVD risk and CVD mortality, with RRs for CVD morbidity being equal to RRs for CVD mortality.
Relative differences in population estimates across IMD quintiles by age and sex group between 1981 and 2030 were equal to relative differences in 2015
Relative differences between ischaemic and haemorrhagic stroke deaths between 2017-2030 were equal to relative differences in 2016
CVD mortality projections using the Bayesian Age-Period-Cohort model assumed that observed age, period, and cohort effects remain the same throughout the modelling period.

Seferidi P, *et al. bmjnph* 2020; 3:e000057. doi: 10.1136/bmjnph-2019-000057

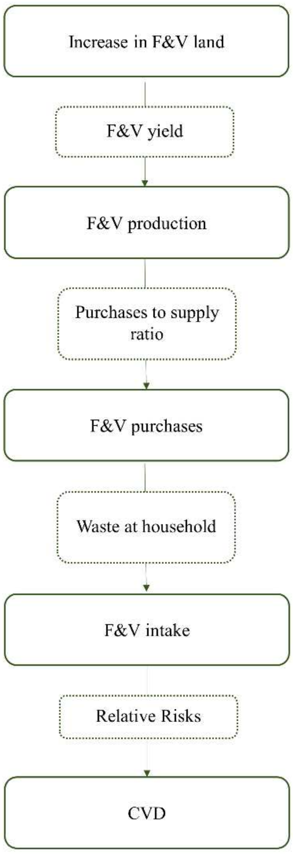

Figure A1. Schematic representation of the model.

Seferidi P, *et al. bmjnph* 2020; 3:e000057. doi: 10.1136/bmjnph-2019-000057

References

1. Bajekal M, Scholes S, Love H, et al. Analysing Recent Socioeconomic Trends in Coronary Heart Disease Mortality in England, 2000-2007: A Population Modelling Study. PLoS Med. 2012;9(6):e1001237. doi:10.1371/journal.pmed.1001237

2. Department for Environment Food & Rural Affairs (DEFRA). Farming Statistics. Final Land Use, Livestock Populations and Agricultural Workforce At 1 June 2017 - England.; 2017. www.statistics.gov.uk. Accessed June 12, 2019.

3. Micha R, Peñalvo JL, Cudhea F, Imamura F, Rehm CD, Mozaffarian D. Association between dietary factors and mortality from heart disease, stroke, and type 2 diabetes in the United States. JAMA. 2017;317(9):912-924. doi:10.1001/jama.2017.0947

4. Department for Environment Food & Rural Affairs (DEFRA). Structure of the agricultural industry in England and the UK at June. https://www.gov.uk/government/statistical-data-sets/structure-of-the-agricultural-industry-in-england-and-the-uk-at-june. Published 2019. Accessed June 8, 2019.

5. Department for Environment Food & Rural Affairs (DEFRA). Agriculture in the United Kingdom data sets. https://www.gov.uk/government/statistical-data-sets/agriculture-in-the-united-kingdom. Published 2018. Accessed June 12, 2019.

6. Department for Environment Food & Rural Affairs (DEFRA). Latest horticulture statistics. https://www.gov.uk/government/statistics/latest-horticulture-statistics. Published 2018. Accessed June 8, 2019.

7. Department for Environment Food & Rural Affairs (DEFRA). Family food datasets. https://www.gov.uk/government/statistical-data-sets/family-food-datasets. Published 2018. Accessed May 12, 2019.

8. WRAP. Household Food and Drink Waste in the United Kingdom 2012.; 2013. www.wrap.org.uk. Accessed June 3, 2019.

9. NatCen Social Research, MRC Elsie Widdowson Laboratory, University College London. Medical School. National Diet and Nutrition Survey Years 1-8, 2008/09-2015/16. 2018. doi:10.5255/UKDA-SN-6533-8

10. Barendregt JJ. The effect size in uncertainty analysis. Value Health. 2010;13(4):388-391. doi:10.1111/j.1524-4733.2009.00686.x

11. Seferidi P, Laverty AA, Pearson-Stuttard J, et al. Impacts of Brexit on fruit and vegetable intake and cardiovascular disease in England: A modelling study. BMJ Open. 2019;9(1):e026966. doi:10.1136/bmjopen-2018-026966
